# Predictive role of serum C-peptide in new-onset renal dysfunction in type 2 diabetes: a longitudinal observational study

**DOI:** 10.3389/fendo.2023.1227260

**Published:** 2023-07-28

**Authors:** Dongmei Sun, Yifei Hu, Yongjun Ma, Huabin Wang

**Affiliations:** Department of Clinical Laboratory, Affiliated Jinhua Hospital, Zhejiang University School of Medicine, Jinhua, Zhejiang, China

**Keywords:** type 2 diabetes, renal dysfunction, serum C-peptide, new-onset, predictive role

## Abstract

**Background:**

Our previous cross-sectional study has demonstrated the independently non-linear relationship between fasting C-peptide with renal dysfunction odds in patients with type 2 diabetes (T2D) in China. This longitudinal observational study aims to explore the role of serum C-peptide in risk prediction of new-onset renal dysfunction, then construct a predictive model based on serum C-peptide and other clinical parameters.

**Methods:**

The patients with T2D and normal renal function at baseline were recruited in this study. The LASSO algorithm was performed to filter potential predictors from the baseline variables. Logistic regression (LR) was performed to construct the predictive model for new-onset renal dysfunction risk. Power analysis was performed to assess the statistical power of the model.

**Results:**

During a 2-year follow-up period, 21.08% (35/166) of subjects with T2D and normal renal function at baseline progressed to renal dysfunction. Six predictors were determined using LASSO regression, including baseline albumin-to-creatinine ratio, glycated hemoglobin, hypertension, retinol-binding protein-to-creatinine ratio, quartiles of fasting C-peptide, and quartiles of fasting C-peptide to 2h postprandial C-peptide ratio. These 6 predictors were incorporated to develop model for renal dysfunction risk prediction using LR. Finally, the LR model achieved a high efficiency, with an AUC of 0.83 (0.76 - 0.91), an accuracy of 75.80%, a sensitivity of 88.60%, and a specificity of 70.80%. According to the power analysis, the statistical power of the LR model was found to be 0.81, which was at a relatively high level. Finally, a nomogram was developed to make the model more available for individualized prediction in clinical practice.

**Conclusion:**

Our results indicated that the baseline level of serum C-peptide had the potential role in the risk prediction of new-onset renal dysfunction. The LR model demonstrated high efficiency and had the potential to guide individualized risk assessments for renal dysfunction in clinical practice.

## 1 Introduction

Diabetes has become a major cause of reduced life expectancy and premature death all over the world (1, 2), and its prevalence is increasing every year (3). Especially when the kidney function is impaired, the mortality of patients with diabetes is obviously increased (4). Unfortunately, approximately 20% to 40% of patients with diabetes may occur diabetic kidney disease (DKD) which is the lead cause of end-stage renal disease (5, 6). DKD is one of the most serious complications of diabetes and has become an important public health issue in most parts of the world (7). Therefore, in order to detect and/or intervene in DKD at an early stage, many research groups are focusing on looking for the clinical risk factors or biomarkers of DKD and constructing predictive models for the risk of occurrence and progression of DKD (8–10).

Up to now, previous studies have recommended some early diagnosis or risk prediction models for DKD based on clinical serum/urine biomarkers of renal function, transcriptomics markers and/or metabonomics markers (11–15). Several clinical risk factors associated with the occurrence and progression of DKD have been reported, including age, gender, hypertension, dyslipidemia, glycated hemoglobin (HbA1c), the drug use of angiotensin converting enzyme inhibitor (ACEI) or angiotensin receptor blocker (ARB), diabetes duration, body mass index (BMI), and smoking, among others (7, 11, 13, 16). Additionally, there have been many studies on urinary biomarkers of renal injury commonly used in clinical practice, such as urinary albumin-to-creatinine ratio (ACR), estimated glomerular filtration rate (eGFR), alpha-1-microglobulin to creatinine ratio (A1MCR), neutrophil gelatinase-associated lipocalin to creatinine ratio (NGAL/Cr), transferrin to creatinine ratio (UTRF/Cr), retinol-binding protein to creatinine ratio (URBP/Cr), and orosomucoid to creatinine ratio (UORM/Cr), among others (7, 13–17).Recently, one of our previous cross-sectional studies has reported that the serum fasting C-peptide is an independent risk factor for the odds of renal dysfunction in patients with type 2 diabetes, furthermore, the association between them is not linear, but non-linear (6). However, the role of serum C-peptide in in predicting the risk of new-onset renal dysfunction remains unclear and needs to be further clarified. Therefore, the present study aims to further explore the role of serum C-peptide in predicting occurrence risk of renal impairment, and construct a predictive model based on serum C-peptide and other clinical parameters for the risk prediction of new-onset renal dysfunction in patients with type 2 diabetes.

## 2 Materials and methods

### 2.1 Study subjects

This was a longitudinal observational study, the subjects of this study were randomly selected from patients with type 2 diabetes who had visited at the Endocrinology Department of the Affiliated Jinhua Hospital, Zhejiang University School of Medicine, between January 2017 and December 2017 (17). All these selected patients had eGFR > 60 mL/min/1.73 m^2^ and ACR <30 mg/g at baseline, and they were advised to return for follow-up appointments every 3-6 months. Finally, the patients underwent a mean 2-year follow-up. The participants were excluded from the present study due to the following exclusion criteria: (1) age < 18 years old at initial phase; (2) patients with renal dysfunction at baseline; (3) patients with serious autoimmune diseases, such as systemic lupus erythematosus; (4) patients with serious infectious diseases, liver diseases and/or tumor; (5) patients who had a rapid renal function decline within 3 months during the follow-up period; (6) patients who lost to follow up; (7) patients with absence of important clinical parameters at baseline, especially the levels of serum C-peptide, 2h postprandial C-peptide. Finally, 166 subjects with type 2 diabetes and normal renal function at baseline were enrolled in the present study. This study was approved by the Ethics Committee of Affiliated Jinhua Hospital, Zhejiang University School of Medicine.

### 2.2 Data extraction

In this study, the demographic features and most of the laboratory parameters at the initial phase were collected from the electronic medical record system, including age, sex, height, weight, hypertension (yes or no), systolic blood pressure (SBP), diastolic blood pressure (DBP), and the use of ACEI/ARB, diabetic duration, serum creatinine, uric acid, fasting C-peptide, 2h postprandial C-peptide, total protein, albumin, total bilirubin, total cholesterol, high-density lipoprotein cholesterol (HDL-C), low-density lipoprotein cholesterol (LDL-C), triglycerides, alanine aminotransferase, aspartate aminotransferase (AST) and HbA1c. During the follow-up period, the first or second urine samples of all these participant at their first and last visits were collected and stored at -80°C, respectively. All the samples were thawed and recentrifuged before measurement. The levels of urinary creatinine, ACR, A1MCR, NGAL/Cr, UTRF/Cr, URBP/Cr and UORM/Cr were detected and calculated by using Beckman Coulter biochemical Analyzer and Byron Diagnostics reagents (Byron, Shanghai, China).

Additionally, the height and weight of the subjects were used to calculate BMI. The serum fasting C-peptide to 2h postprandial C-peptide ratio was defined as C0/C2. The levels of eGFR were obtained using the algorithm of Xiangya equation (18). In the present study, ACR > 30 mg/g and/or eGFR < 60 ml/min/1.73m^2^ were defined as renal dysfunction.

### 2.3 Statistical analyses

All the statistical analyses in this study were conducted by using the Deepwise and Beckman Coulter DxAI platform (https://dxonline.deepwise.com/) and R software (4.2.3 version). The normal distribution variables were presented as mean ± standard deviation (SD); the continuous variables with skewed distribution were presented as median (Q1 - Q3); the categorical variables were presented as frequencies with percentages. Pearson’s chi-squared test was used to compare the differences of the categorical variables between subjects with and without new onset renal dysfunction during follow-up period. Unpaired t-test and Mann-Whitney test were performed to conduct the between-group comparisons of continuous variables, as appropriate. Spearman correlation analyses were executed to evaluate the linear relationships between the continuous variables. By the principle of avoiding multicollinearity and over-fitting of the model, the least absolute shrinkage and selection operator (LASSO) regression was performed to filtrate the potential predictors (seed = 1). In the present study, predictors were chosen according to the lambda value with the minimum mean square error. Of note, according to the findings of the previous study, fasting C-peptide and UA were non-linearly associated with the odds of renal dysfunction, therefore, the quartiles of fasting C-peptide, C0/C2 and UA were also analyzed as the potential variables in LASSO regression, respectively.

Logistic regression (LR) was used to establish the predictive model. 5-fold cross-validation was used to validate the stability of the models. The area under the receiver operating characteristic curve (AUC), accuracy, sensitivity and specificity were used to assess the performance of the model. The Shapley additive explanations (SHAP) values were also analyzed to evaluate the importance of every potential predictor. Power analysis was performed to assess the statistical power of the model. A two-tailed p-value < 0.05 was considered statistically significant.

## 3 Results

### 3.1 Baseline characteristics of the participants

At the initial phase of this study, 166 subject with type 2 diabetes and normal renal function were included, 61.45% (102) of them were male, 38.55% (64) were female, 39.76% (66) had hypertension, the average age was 57.11 ± 12.31 years old, and the median follow-up time was 22.35 months. During the 2-year follow-up, 21.08% (35/166) of participants progressed to renal dysfunction. **Table 1** listed the baseline clinical characteristics of the participants. The baseline levels of HbA1c, ACR, A1MCR, UTRF/Cr, URBP/Cr and UORM/Cr presented significantly difference between subjects with and without renal dysfunction development (p < 0.05). However, no statistical differences between these two groups were detected for other clinical features. Of note, although the prevalence of hypertension did not show the statistical difference between the two groups, the proportion of hypertension was higher in participants with renal impairment development compared with the subjects without renal impairment development (51.43% vs 36.64%).

**Table 1:** The baseline clinical characteristics of the participants.

Clinical variables	Overall (n=166)	Subjects without renal impairment development (n=131)	Subjects with renal impairment development (n=35)	p*
Age, years	57.11 ± 12.31	56.59 ± 11.71	59.09 ± 14.19	0.289
BMI, kg/m^2^	24.31 ± 3.27	24.29 ± 3.37	24.38 ± 2.89	0.878
Male, n(%)	102 (61.45)	83 (63.36)	19 (54.29)	0.327
SBP, mmHg	133 ± 18	133 ± 17	134 ± 18	0.786
DBP, mmHg	78 ± 10	78 ± 10	79 ± 10	0.657
Hypertension,n (%)	66 (39.76)	48 (36.64)	18 (51.43)	0.112
ACEI/ARB use,n (%)	45 (27.10)	35 (26.72)	10 (28.57)	0.827
Diabetic duration, years	8.56 ± 5.68	8.8 1± 5.70	7.64 ± 5.51	0.282
Total cholesterol, mmol/L	4.54 ± 1.14	4.51 ± 1.15	4.67 ± 1.10	0.441
Triglyceride, mmol/L	2.14 ± 2.60	2.12 ± 2.51	2.20 ± 2.91	0.868
HDL, mmol/L	1.20 ± 0.27	1.19 ± 0.27	1.22 ± 0.28	0.553
LDL, mmol/L	2.98 ± 0.83	2.95 ± 0.84	3.12 ± 0.82	0.276
HbA1c, %	8.52 ± 2.20	8.20 ± 2.02	9.71 ± 2.44	0.002
Serum creatinine, μmol/L	74.24 ± 12.42	74.49 ± 12.53	73.31 ± 11.98	0.622
UA, μmol/L	298.31 ± 66.74	302.70 ± 67.46	281.86 ± 61.26	0.100
AST, U/L	23.75 ± 13.64	24.13 ± 14.56	22.34 ± 9.28	0.493
Fasting C-peptide, nmol/L	0.41 [0.25, 0.61]	0.40 [0.23, 0.60]	0.44 [0.30, 0.69]	0.412
2h C-peptide, nmol/L	1.27 [0.57, 1.88]	1.23 [0.56, 1.78]	1.33 [0.60, 2.05]	0.351
C0/C2	0.37 [0.24, 0.57]	0.39 [0.24, 0.60]	0.37 [0.24, 0.51]	0.78
Urinary creatinine, μmol/L	7517 [5258, 11430]	7695 [5231, 11197]	6548 [5319, 15182]	0.929
ACR, mg/g	12.87 [7.52, 19.36]	11.91 [6.70, 16.76]	19.22 [12.39, 24.16]	<0.001
eGFR, ml/min/1.73 m^2^	78.37 ± 8.79	78.58 ± 8.47	77.56 ± 9.83	0.544
URBP/Cr, mg/g	0.14 [0.07, 0.32]	0.13 [0.06, 0.27]	0.26 [0.11, 0.45]	0.004
UTRF/Cr, mg/g	0.41 [0.12, 0.96]	0.36 [0.10, 0.79]	0.71 [0.34, 1.13]	0.004
NGAL/Cr, μg/g	16.80 [7.02, 44.92]	14.96 [6.38, 39.94]	26.11 [9.57, 70.17]	0.122
A1MCR, mg/g	7.22 [2.16, 15.76]	6.53 [2.00, 14.02]	9.90 [5.59, 22.30]	0.008
UORM/CR, mg/g	1.49 [0.47, 4.23]	1.25 [0.39, 3.75]	3.57 [1.49, 5.66]	<0.001

*The comparisons between participants with and without renal impairment development. SBP, systolic blood pressure; DBP, diastolic blood pressure; ACEI, angiotensin-converting enzyme inhibitor; ARB, angiotensin receptor blocker; HDL-C, high-density lipoprotein cholesterol; LDL-C, low-density lipoprotein cholesterol; HbA1c, glycated hemoglobin; UA, uric acid; AST, aspartate aminotransferase; C0/C2, fasting C-peptide to 2h C-peptide ratio; ACR, albumin to creatinine ratio; eGFR, estimated glomerular filtration rate; URBP/Cr, retinol-binding protein to creatinine ratio; UTRF/Cr, transferrin to creatinine ratio; NGAL/Cr, neutrophil gelatinase-associated lipocalin to creatinine ratio; UORM/Cr, alpha-1-acid-glycoprotein to creatinine ratio; A1MCR, alpha-1-microglobulin to creatinine ratio.

### 3.2 The analyses of linear correlation between the continuous features

**Figure 1:**
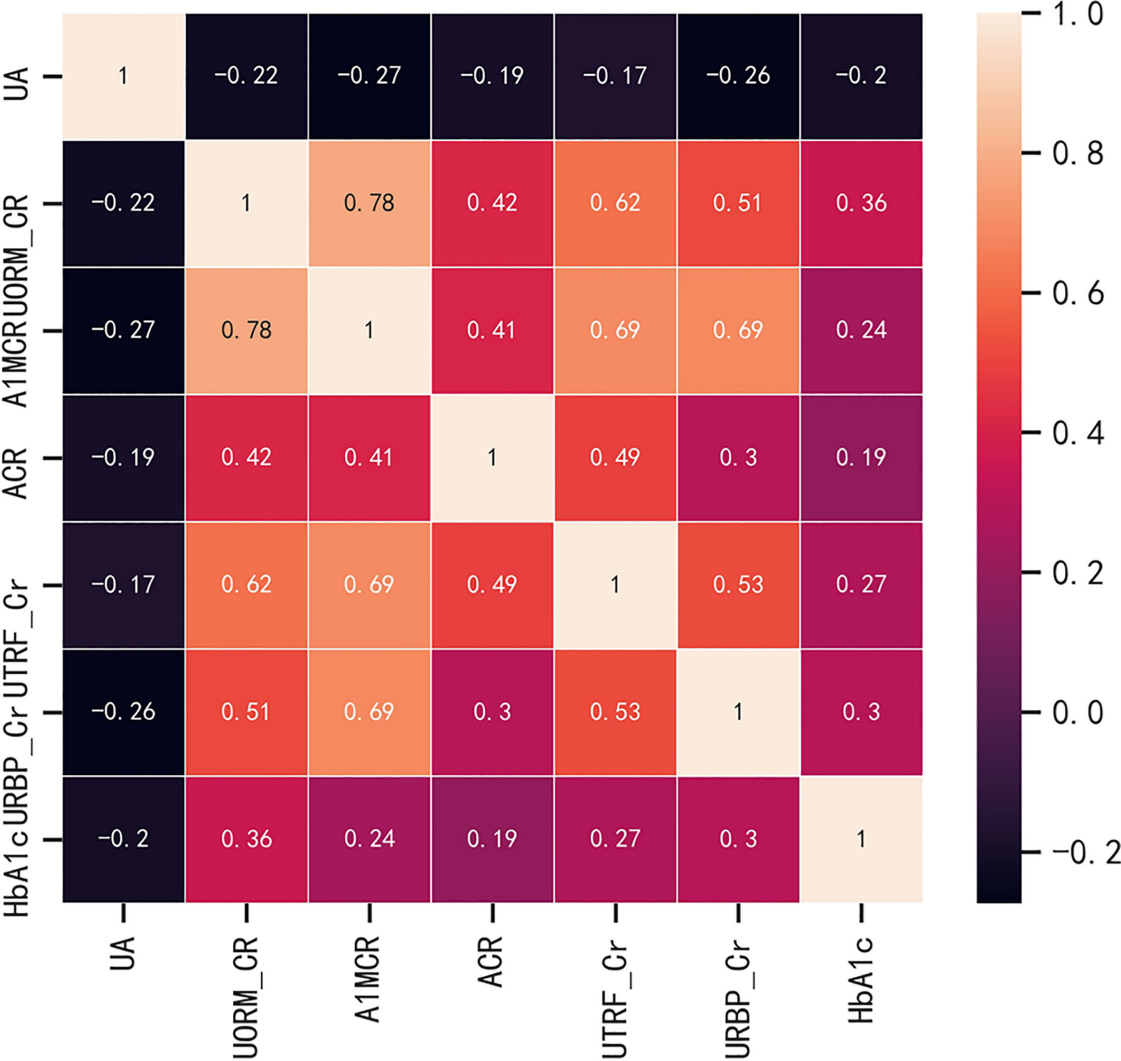
The correlations between the potential continuous variables for the univariate analysis (p ≤ 0.1). HbA1c, glycated hemoglobin; UA, uric acid; ACR, albumin to creatinine ratio; URBP/Cr, retinol-binding protein to creatinine ratio; UTRF/Cr, transferrin to creatinine ratio; UORM/Cr, alpha-1-acid-glycoprotein to creatinine ratio; A1MCR, alpha-1-microglobulin to creatinine ratio.

Before the construction of the predictive models, in order to evaluate the multicollinearity, the linear correlations between the potential continuous variables for the univariate analysis (p ≤ 0.1) in **Table 1** were analyzed, including the basal levels of HbA1c, UA, ACR, A1MCR, UTRF/Cr, URBP/Cr and UORM/Cr (**Figure 1**). According to the heat map, UA presented poor relationships with other variables, however, there was a certain degree of correlation between the other variables. In especial, A1MCR and UORM/Cr (r = 0.78), URBP/Cr and A1MCR (r = 0.69) had the relatively highest correlation, indicating that the differential variables between the two groups could not be used to establish the predictive models directly due to the multicollinearity.

### 3.3 Potential predictor filtration using LASSO regression

**Figure 2:**
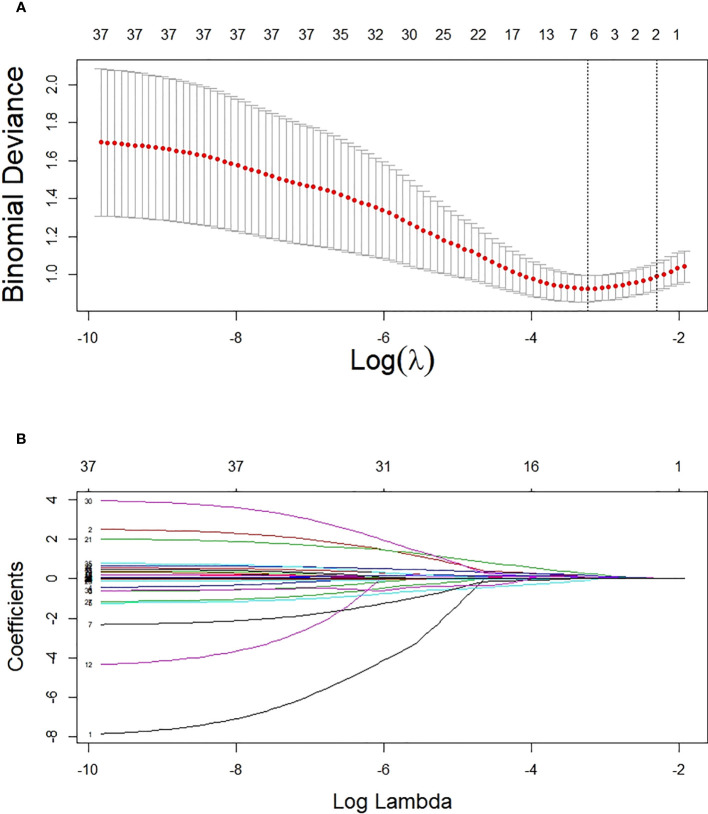
Potential predictor selection using the LASSO regression method. **(A)** Tuning parameter selection in the LASSO regression used 10-fold cross-validation. **(B)** LASSO regression coefficient profiles of variables. In the present study, predictors were chosen according to the lambda value with the minimum mean square error (seed = 1).

Based on non-zero coefficients corresponding to the lambda value with the minimum mean square error in the LASSO regression analysis (seed = 1), six predictors were determined from 34 basal clinical parameters, including ACR, HbA1c, hypertension (yes or no), URBP/Cr, fasting C-peptide quartiles, and C0/C2 quartiles (**Figure 2**).

### 3.4 Construction and validation of the predictive model

**Figure 3:**
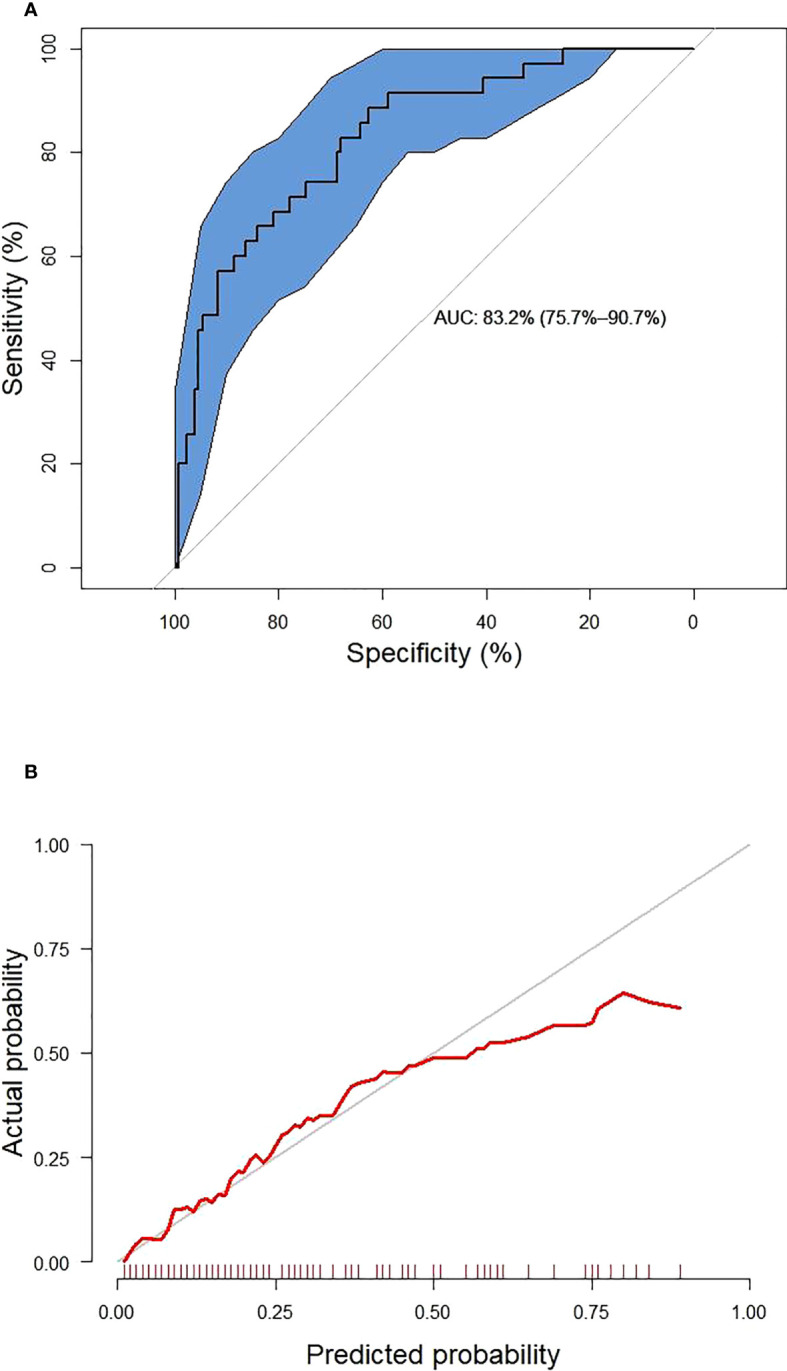
The receiver operating characteristic curve **(A)** and calibration curve **(B)** of the established model. The blue area represents 95% confidence interval.

**Figure 4:**
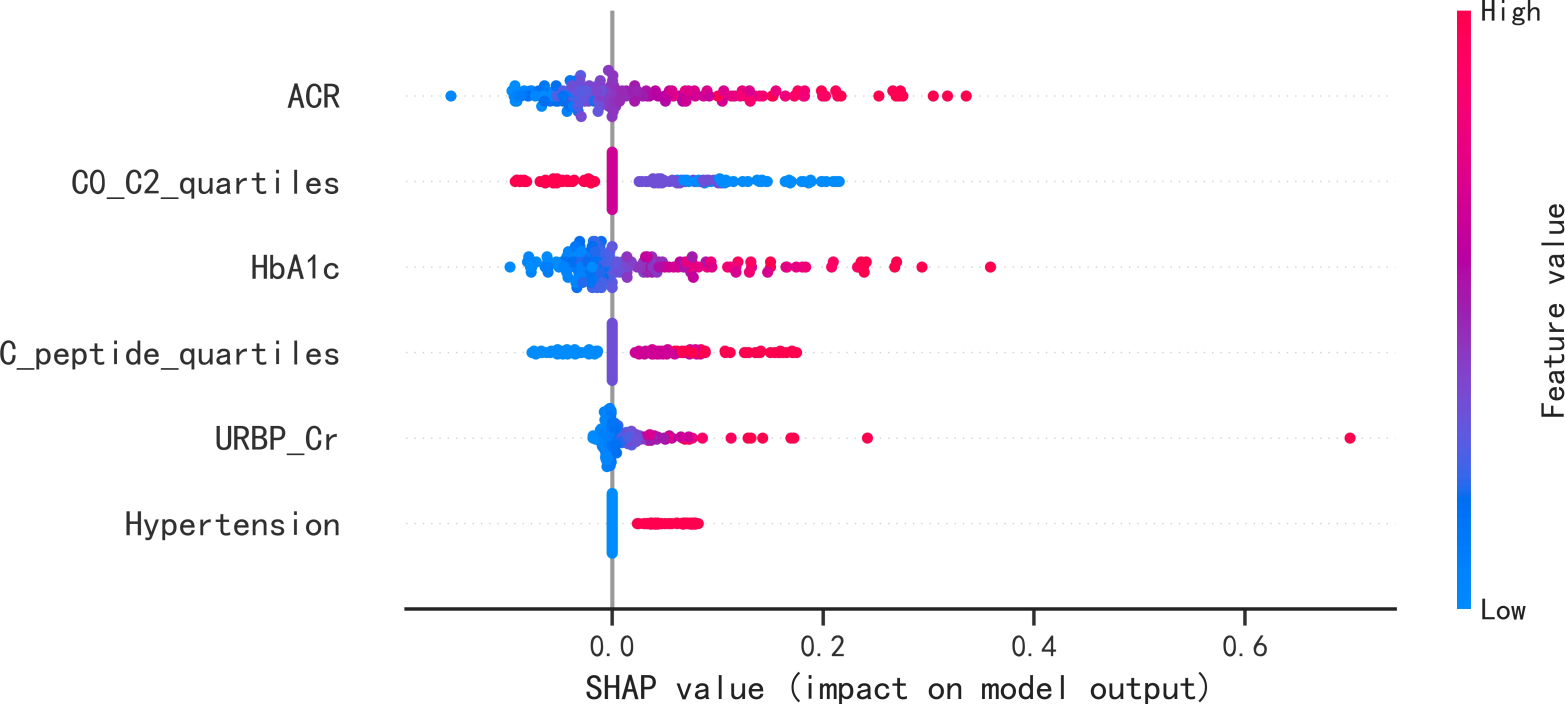
SHAP summary plots of the six predictors for new onset renal dysfunction risk in LR model. SHAP, Shapley additive explanations; ACR, albumin to creatinine ratio; HbA1c, glycated hemoglobin; C0/C2, fasting C-peptide to 2h C-peptide ratio; URBP/Cr, retinol-binding protein to creatinine ratio.

These six predictors selected out by LASSO regression were combined to establish the prediction model for new onset renal dysfunction risk using LR algorithm. **Figure 3** showed the ROC curve and calibration curve of this model. On the whole, the model demonstrated a high prediction performance, with an AUC of 0.83 (0.76 - 0.91), an accuracy of 75.80%, a sensitivity of 88.60%, and a specificity of 70.80%; additionally, the calibration curve demonstrated good consistency between observed and predicted outcomes (P > 0.05). The validation of the model was performed using 5-fold cross-validation method. The accuracy of this model was 0.85 ± 0.016 in the training sets, 0.80 ± 0.094 in the test sets, indicating a good capability for assessing renal dysfunction risk. The importance of the predictors was arranged as follows in a descending order: ACR, C0/C2 quartiles, HbA1c, fasting C-peptide quartiles, URBP/Cr and hypertension (**Figure 4**).

### 3.5 Visualization of the optimal prediction model

**Figure 5:**
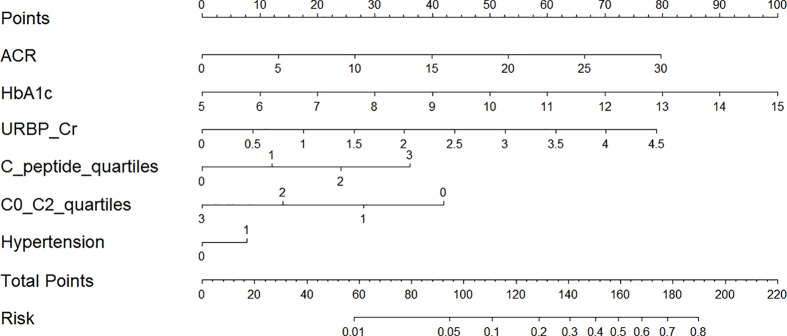
Nomogram for prediction of new-onset renal dysfunction risk based on the LR model. ACR, albumin to creatinine ratio; HbA1c, glycated hemoglobin; C0/C2, fasting C-peptide to 2h C-peptide ratio; URBP/Cr, retinol-binding protein to creatinine ratio. Hypertension 0: patients without hypertension; Hypertension 1: patients with hypertension; C-peptide quartiles 0-3: quartile 1, 2, 3 and 4 of fasting C-peptide; C0/C2 quartiles 0-3: quartile 1, 2, 3 and 4 of C0/C2.

After conducting power analysis, the power of the LR model for predicting the risk of renal impairment was found to be 0.81, which was considered a relatively high level of statistical power. A nomogram graph was developed based on the LR model to make the model more available for individualized prediction of new onset renal impairment risk in clinical practice. For each individual, the values of various covariates corresponded to the Points, respectively; and the total points accumulated by the points of various covariates corresponded to the risk of new onset renal dysfunction (**Figure 5**).

## 4 Discussion

In the present study, in order to avoid multicollinearity and the over-fitting of the model, LASSO regression was performed to screen out six potential predictors from 34 clinical variables, including ACR, HbA1c, URBP/Cr, fasting C-peptide quartiles, C0/C2 quartiles and hypertension. Using these predictors, we developed and validated LR algorithm to predict the new-onset risk of renal dysfunction in patients with type 2 diabetes. Generally, the LR model provided a high performance. Furthermore, based on power analysis, the statistical power of the LR model used to predict the risk of kidney injury was calculated to be 0.81, which corresponded to a relatively high level of statistical power. This suggested that this model had a strong ability to detect true relationships between the predictor variables and the outcome of interest, therefore, we concluded that this model had reliable and valid statistical efficacy. We further constructed a nomogram graph to make the model more available for individualized prediction of new onset renal impairment risk. It is noteworthy that serum C-peptide in the forms of fasting C-peptide quartiles and C0/C2 quartiles played an important role in the model. To our knowledge, this is the first study which clarified the potential role of serum C-peptide in predicting the occurrence risk of renal dysfunction in patients with type 2 diabetes.

Chronic kidney disease is one of the most common complication of diabetes. Due to the high prevalence of diabetes all over the world, the population with type 2 diabetes and chronic kidney disease is increasing rapidly every year, and this has become a global healthcare burden (19, 20). Identifying patients with type 2 diabetes at high risk of renal impairment or chronic kidney disease has the potential value to improve clinical outcome (21). Therefore, many research groups have tried to investigate the clinical risk factors associated with kidney impairment and develop the models to predict the risk of occurrence and/or development of DKD in patients with type 2 diabetes (11, 13, 22). Jiang et al. (11) screened out 9 potential predictors based on a systematic review and meta-analysis of 20 cohorts, then establish a prediction model for detecting patients at high risk of DKD. Chan and Damrauer et al. (22) developed a prognostic risk score (KidneyIntelX™) combining electronic health records and laboratory biomarkers to predict the progression of DKD using supervised random forest algorithm. Xu et al. (13) used the LR regression to determine the potential predictors and develop a model for predicting the short-term risk of renal dysfunction occurrence in patients with type 2 diabetes. However, none of these previously established models included serum C-peptide, and the potential value of C-peptide in predicting the risk of renal injury in patients with type 2 diabetes was not mentioned (11, 13, 22), therefore, building upon our previous research (6), this study aimed to explore the potential role of serum C-peptide in predicting the risk of new-onset renal dysfunction in patients with type 2 diabetes.

In the present study, six predictive features were determined by LASSO regression, and LR algorithms was performed to establish the prediction model for the occurrence of renal dysfunction. The study by Hosseini Sarkhosh et al. (23) determined six predictors (ACR, HbA1c, hypertension, diabetic duration, eGFR and cardiovascular disease) using recursive feature elimination with cross-validation and develop a risk model with a AUC of 0.755 for predicting renal dysfunction using multivariate LR algorithm. By comparison, the AUC in the present study was a little higher than that in the study of Hosseini Sarkhosh et al. (23), although both of the two studies had six predictive features. Perhaps, the most likely reasons for the different performance of the prediction models in the two studies were the study population and the predictive variables. In the present study, the serum C-peptide in terms of fasting C-peptide quartiles and C0/C2 quartiles was an important predictor in the established model. C-peptide is recognized as a reliable indicator of the function of pancreatic β-cell, and it is considered as an inert molecule in the past (24). Recently, the multiple functions of C-peptide such as signaling pathways activation, physiological effects, and protection against complications of diabetes has been gradually discovered (6, 25). In a real world study conducted by Huang et al. (26), it was demonstrated that the study subjects with 1.71 ≤ C-peptide < 2.51 ng/mL had a higher glycemic control rate among the population with type 2 diabetes. A previous study of our research group demonstrated the potential role of the fasting C-peptide in odds stratification of renal dysfunction in type 2 diabetes (6). However, the role of serum C-peptide in risk prediction of new-onset renal dysfunction remained unclear. Therefore, as a clinical feature, the association of serum C-peptide with the occurrence risk of renal impairment was analyzed in this study. Although no statistical difference of fasting C-peptide, 2h postprandial C-peptide and C0/C2 were detected between the participants with and without new onset renal dysfunction, the fasting C-peptide quartiles and C0/C2 quartiles were screened out by LASSO regression as the high-ranking predictors in the present study. In addition, according to the SHAP summary plots, the impact of C0/C2 quartiles in the LR model second only to the impact of ACR, indicating that the C0/C2 quartiles played an important role in predicting renal impairment risk.

The highlight of this study included that in order to avoid the multicollinearity and the over-fitting of the models, LASSO regression was performed to filter the potential predictors; and the power analysis validated the statistical power and reliability of this model, suggested that it had a strong ability to detect true relationships between the predictor variables and the outcome of interest. Moreover, to our knowledge, we firstly clarified the potential role of serum C-peptide in predicting the occurrence risk of renal dysfunction in patients with type 2 diabetes. However, several limitations of the present study were worth noting. First, due to lack of complete information of fasting C-peptide, 2h postprandial C-peptide levels in many patients, the sample size of the longitudinal observational study was relatively small, and the participants were recruited from a single center, so the findings might be lack of generalizability, potentially limiting its application. Second, an external validation was needed to validate the prediction performance of the LR model. Thus, more prospective, multicenter and larger sample-size studies were needed to elucidate these findings.

In conclusion, we demonstrated that serum C-peptide had a potential role in predicting the new onset renal dysfunction in patients with type 2 diabetes. The LR model had a high performance for renal dysfunction risk prediction. The nomogram graph based on the LR model might provide an available tool for individualized prediction of new onset renal impairment risk. Generally, this study is a continuation of our previous cross-sectional study (6), and it paves the way for the need to design and execute a prospective confirmatory study in a random sample with the proper dimension.

## Data availability statement

The raw data supporting the conclusions of this article will be made available by the authors, without undue reservation.

## Ethics statement

The studies involving human participants were reviewed and approved by Ethics Committee of Affiliated Jinhua Hospital, Zhejiang University School of Medicine. The patients/participants provided their written informed consent to participate in this study.

## Author contributions

Study design: DS, YM, HW. Statistical analysis: HW, YH,YM. Manuscript writing: DS, HW. Data collection: DS, YH. All authors contributed to the article and approved the submitted version.
